# Pharmacogenetics driving personalized medicine: analysis of genetic polymorphisms related to breast cancer medications in Italian isolated populations

**DOI:** 10.1186/s12967-016-0778-z

**Published:** 2016-01-22

**Authors:** Massimiliano Cocca, Davide Bedognetti, Martina La Bianca, Paolo Gasparini, Giorgia Girotto

**Affiliations:** Department of Medical, Surgical and Health Sciences, University of Trieste, Trieste, Italy; Tumor Biology, Immunology, and Therapy Section, Division of Translational Medicine, Sidra Medical and Research Center, Doha, Qatar; IRCCS Burlo Garofolo, Trieste, Italy; Division of Experimental Genetics, Sidra Medical and Research Center, Doha, Qatar

## Abstract

**Background:**

Breast cancer is the most common cancer in women characterized by a high variable clinical outcome among individuals treated with equivalent regimens and novel targeted therapies. In this study, we performed a population based approach intersecting high-throughput genotype data from Friuli Venezia Giulia (FVG) isolated populations with publically available pharmacogenomics information to estimate the frequency of genotypes correlated with responsiveness to breast cancer treatment thus improving the clinical management of this disease in an efficient and cost effective way.

**Methods:**

A list of 80 variants reported to be related to the efficacy or toxicity of breast cancer drugs was obtained from PharmGKB database. Fourty-one were present in FVG, 1000G European (EUR) and ExAC (Non Finnish European) databases. Their frequency was extracted using PLINK software and the differences tested by Fisher’s exact test.

**Results:**

Statistical analyses revealed that 13 out of the 41 (32 %) variants were significantly different in frequency in our sample as compared to the EUR/ExAC cohorts. For nine variants the available level of evidence (LOE) included polymorphisms related to cyclophosphamide, tamoxifen, doxorubicin, fluorpyrimidine and paclitaxel. In particular, for trastuzumab two variants were detected: (1) rs1801274-G within *FCGR2A* and associated with decreased efficacy (LOE 2B); (2) rs1136201-G located within *ERBB2* and associated with increased toxicity (LOE 3). Both these two variants were underrepresented in the FVG population compared to EUR/ExAC population thus suggesting a high therapeutic index of this drug in our population. Moreover, as regards fluoropyrimidines, the frequency of two polymorphisms within the *DPYD* gene associated with drug toxicity (e.g., rs2297595-C allele and rs3918290-T allele, LOE 2A and 1, respectively) was extremely low in FVG population thus suggesting that a larger number of FVG patients could benefit from full dosage of fluoropyrimidine therapy.

**Conclusions:**

All these findings increase the overall knowledge on the prevalence of specific variants related with breast cancer treatment responsiveness in FVG population and highlight the importance of assessing gene polymorphisms related with cancer medications in isolated communities.

**Electronic supplementary material:**

The online version of this article (doi:10.1186/s12967-016-0778-z) contains supplementary material, which is available to authorized users.

## Background

The development of refined technologies for genetic analysis (e.g., next-generation sequencing, genotyping, etc.), paired with a continuous optimization of computational and bioinformatic tools, has recently unveiled the scope of human genetic variations. These high-throughput approaches led to the discovery of novel disease-associated variants, germline mutations responsible for rare genetic diseases, and, as far as the cancer field is concerned, to the identification of somatic mutations predictive of treatment responsiveness [1]. The characterization of patient-specific genetic make up is critical for the development of personalized interventions. A medication that is proven efficacious in many patients, often fails to work in others. Furthermore, even if a certain drug is active, it still may cause serious side effects [2]. Pharmacogenomics addresses this issue by seeking to identify genetic contributors to human variation in drug efficacy and toxicity with the hope of developing personalized treatments.

Breast cancer is the most common cancer in women worldwide. An early detection combined with an appropriate treatment has proved to be effective in reducing risk of death and relapse [3–5]. Nevertheless, in the adjuvant setting, only few patients will actually benefit from the treatment. Similarly, a wide degree of variation in treatment sensitivity is observed in metastatic setting.

There is a tremendous effort to identify factors associated with treatment responsiveness [6–8]. Although most of the studies have been focusing on tumor characteristics, it is clear that host’s genetic make up can influence treatment tolerability and outcome.

The effect of several major antineoplastic agents is influenced by genetic polymorphisms of different nature ranging from the target itself (e.g., transtuzumab and *HER2* [9] to metabolic pathways (e.g., capecitabine and *DPYD*) [10]. In breast cancer, as for other diseases, there is high degree of heterogeneity in term of clinical outcome among individuals treated with equivalent regimens such as hormonal agents (e.g., tamoxifen), cytotoxic agents (e.g., capecitabine), and targeted therapies [11].

In view of this heterogeneity, an increased awareness of the distribution of risk variants within a specific population (or community) is critical to plan tailored health-care interventions [12]. At the best of our knowledge, many studies described drug response related to the variations in the general population but none of them have so far analyzed the prevalence of defined risk alleles (i.e., variants associated with treatment toxicity or treatment failure) in isolated communities such as those ones described below. In fact, the detection of an unusual high rate of one or more risk variants in a defined community could prompt the local authority to implement ad-hoc screening strategies, which wouldn’t be otherwise cost-effective in the general population. In addition, physicians can use this information to better sharpen the risk–benefit ratio of a specific intervention in every-day clinical practice.

Here, we developed an analytic pipeline that intersects high-throughput genotype data with publically available pharmacogenomics information. Through this approach, we described the frequency of genetic markers correlated with responsiveness to breast cancer treatment in the Friuli Venezia Giulia (FVG) population with the aim of improving clinical management of this disease in a cost-effective way. FVG is an autonomous region located in North Eastern Italy at the border with Austria and Slovenia constituted by 1.2 million of inhabitants. Thanks to this autonomy and to the effective clinical data exchange between different health service providers, FGV has developed a regional health care network (RHCN) ensuring continuity of care and improved health services for citizens of this region. This exchange is facilitated by an information technology (IT) infrastructure that allows a continuous and up-to-date secure exchange of medical data and records. As of 2008, medical data from hospitals, primary care physicians, and pharmacies (digital prescription records) are all accessible through this IT infrastructure in a strictly regulated manner.

Moreover, in 2009, two population based research pilot projects have been started. One is the FVG genetic park, focused in studying six isolated communities of this region (Erto-Casso, Clauzetto, Resia, Sauris, San Martino del Carso, and Illegio) for an overall number of approximately 2500 inhabitants [13]. The second one is the MoMa, which aims at investigating dismetabolic syndromes in another large community of the region (Montereale/Maniago, approx. 15,000 inhabitants). Both volunteer-based projects, representing approximately 2 % of the adult population living in the FVG autonomous region, have established a biobank which stores biological samples and a huge collection of clinical data. Taken together, the existence of all the elements mentioned above sets FVG Region in a globally unique position for the implementation of genomic medicine.

## Methods

### Sample collection, DNA sampling and genotyping

**Fig. 1:**
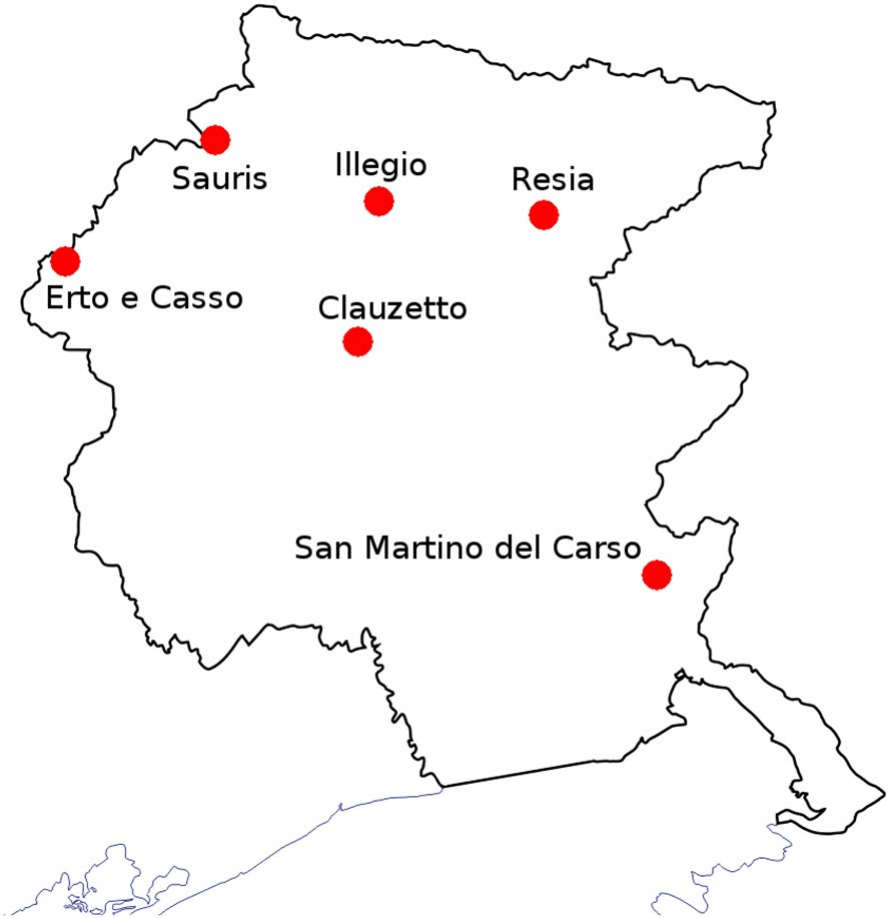
Friuli Venezia Giulia villages. Geographic location of the six isolated villages in the Friuli Venezia Giulia region (North Eastern Italy) analyzed in this study. The overall number of recruited people is 1590

**Fig. 2:**
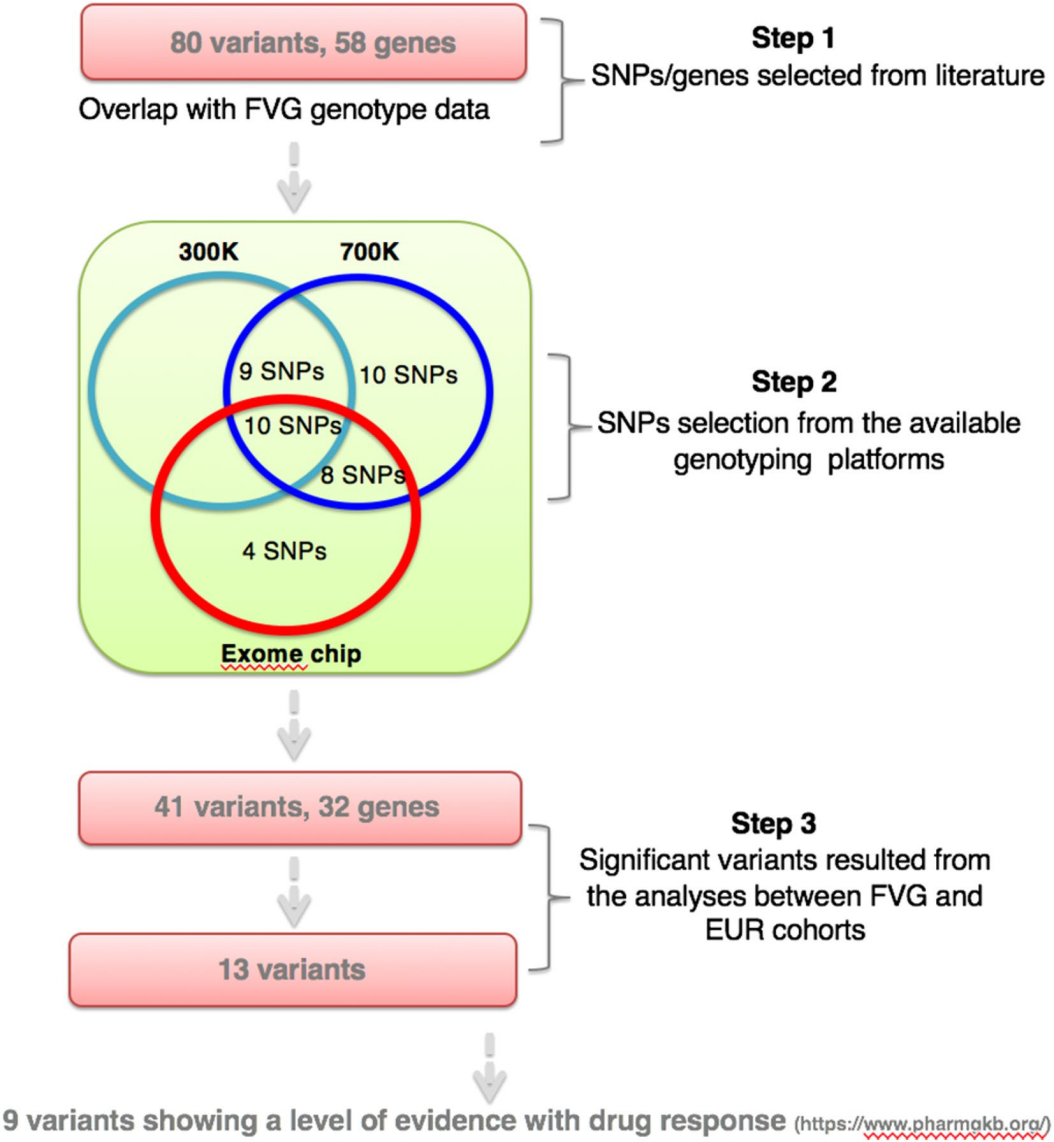
Pipeline used for variants’ selection. Three steps were carried out: Step (1) 80 variants and 58 genes related to breast cancer medicationsa ccording to the PharmGKB database were considered. Step (2) Variants selected in the previous step were overlapped with data from the available genotyping platforms in the FVG cohort: 41 variants and 32 genes were used for further analysis. Step (3) Frequencies for the 41 variants selected in step 2 were compared between FVG and EUR cohorts, resulting in a set of 13 variants. Among them, for nine variants the association was supported by a certain LOE

One thousand five hundred ninety samples from the FGV genetic park (six isolated villages, see Fig. 1) project were used for our genomic analyses (Additional file 1: Table S1) [13]. Information from a standardized health examination with collection of a series of deep phenotypes (neurological, psychiatric, audiological, ophtalmological, cardiovascular, etc.) and a questionnaire on health-related topics, such as lifestyle and diet were collected from all participants. The exact number of participants divided by village, together with information about sex and mean age are reported in Additional file 1: Table S1. DNA from blood samples was extracted using standard protocols. All the 1590 subjects were genotyped using the HumanExome BeadChip. In addition, a subset of 1259 individuals has been genotyped using the Illumina HumanCNV370-Quadv3_C (300 K), and another subset (N = 331) with the Illumina HumanOmniExpress-12v1-Multi_C chip (700 K) (see Fig. 2). Genotype quality control and data cleaning were performed as previously described [13]. Considering the different genotyping platforms, the number of samples available for the analysis of each variant ranged from 331 to 1590 (the vast majority of cases) as described in Table 1. All participants have signed a broad informed consent form (a 5 years follow-up is now in progress), which allows the continuous updating of epidemiological data through periodical linking to National electronic databases and registries. The research was conducted according to the ethical standards defined by the Helsinki declaration. The study was approved by the Institutional Review Board of IRCCS Burlo Garofolo PROT CE/v-78.

**Table 1:** Alleles frequencies among FVG, 1000G and ExAC (Non Finnish European population) data of a selected list of SNPs reported as associated with specific drugs in the PharmGkB database

Snp id (gene)	Drugs (reported as related by PharmGKB database)	Alleles	Af_FVG	Af_1000G_EUR	Af_ExAC_NFE	Samples	p value vs EUR	p value vs ExAC
*rs3918290* (*DPYD*)	*Capecitabine*	*T*/C	*0.0006325*	*0.01*	*0.0058*	*1581*	*2.062E−05*	*2.20E−04*
*rs1136201* (*ERBB2*)	*Trastuzumab*	*G*/A	*0.1692*	*0.25*	*0.2396*	*1581*	*3.562E−05*	*2.37E−13*
*rs2369049* (*TCL1A*)	*Exemestane*	*G*/A	*0.1111*	*0.17*	*NA*	*1580*	*1.411E−04*	*NA*
*rs4244285* (*CYP2C19*)	*Doxorubicin, cyclophosphamide, tamoxifen*	*A*/G	*0.08485*	*0.15*	*0.1477*	*330*	*1.077E−03*	*7.27E−05*
*rs714368* (*SLC22A16*)	*Doxorubicin, doxorubicinol*	*C*/T	*0.2726*	*0.21*	*0.2218*	*1590*	*6.932E−03*	*1.73E−07*
*rs776746* (*CYP3A5*)	*Tamoxifen*	*T*/C	*0.08055*	*0.05*	*NA*	*1589*	*8.936E−03*	*NA*
*rs1801274* (*FCGR2A*)	*Trastuzumab, doxorubicin, paclitaxel, cyclophosphamide*	*G*/A	*0.4195*	*0.5*	*0.4942*	*1590*	*1.457E−02*	*9.20E−07*
*rs4880* (*SOD2*)	*Cyclophosphamide*	*G*/A	*0.5452*	*0.46*	*0.5162*	*1581*	*1.877E−02*	*7.81E−02*
*rs1801159* (*DPYD*)	*Capecitabine*	*C*/T	*0.217*	*0.17*	*0.1978*	*1581*	*2.155E−02*	*3.36E−02*
*rs2072671* (*CDA*)	*Capecitabine*	*C*/A	*0.397*	*0.33*	*0.3427*	*1587*	*2.326E−02*	*1.79E−05*
*rs2297595* (*DPYD*)	*Fluorouracil, capecitabine*	*C*/T	*0.09077*	*0.12*	*0.1027*	*1581*	*3.256E−02*	*5.40E−02*
*rs1048943* (*CYP1A1*)	*Docetaxel, capecitabine*	*C*/T	*0.06839*	*0.04*	*0.0332*	*1581*	*2.374E−02*	*5.66E−18*
*rs6214* (*IGF1*)	*Tamoxifen*	*T*/C	*0.3518*	*0.41*	*NA*	*1589*	*4.746E−02*	*NA*
rs1695 (GSTP1)	Docetaxel, epirubicin, fluorouracil, doxorubicin, cyclophosphamide	*G*/A	0.2761	0.32	0.3191	1590	8.264E−02	2.11E−04
rs12210538 (SLC22A16)	Doxorubicin, cyclophosphamide	*G*/A	0.2125	0.25	0.2351	1581	8.411E−02	2.11E−02
rs9024 (CBR1, SETD4)	Doxorubicin, doxorubicinol	*A*/G	0.1243	0.1	NA	1585	1.116E−01	NA
rs7349683 (EPHA5)	Paclitaxel	*T*/C	0.4169	0.37	0.3588	1589	1.312E−01	8.26E−06
rs45589337 (DPYD)	Cyclophosphamide, fluorouracil, methotrexate	*C*/T	0.004744	0.01	0.0155	1581	1.504E−01	2.90E−06
rs10509373 (C10orf11)	Tamoxifen	*C*/T	0.469	0.42	NA	1258	1.544E−01	NA
rs1056836 (CYP1B1)	Docetaxel, paclitaxel, taxanes	*G*/C	0.5728	0.63	0.5675	1580	1.551E−01	7.69E−01
rs723685 (SLC22A16)	Doxorubicin, cyclophosphamide	*G*/A	0.1069	0.09	0.0886	1590	2.396E−01	1.28E−03
rs1143684 (NQO2)	Doxorubicin, cyclophosphamide	*T*/C	0.7434	0.8	0.787	1588	2.401E−01	4.18E−02
rs9561778 (ABCC4)	Doxorubicin, cyclophosphamide, fluorouracil	*T*/G	0.2085	0.18	NA	331	2.943E−01	NA
rs2032582 (ABCB1)	Anthracyclines and related substances, paclitaxel, doxorubicin, cyclophosphamide, taxanes	*C*/A	0.6091	0.57	0.5523	1581	3.374E−01	9.99E−04
rs8133052 (CBR3)	Doxorubicin	*A*/G	0.4817	0.45	0.5904	1587	3.647E−01	5.46E−09
rs2290272 (SLC28A1)	Capecitabine	*A*/G	0.3569	0.38	0.3466	1590	4.403E−01	4.20E−01
rs1128503 (ABCB1)	Doxorubicin	*G*/A	0.5317	0.57	0.5726	331	4.631E−01	2.78E−01
rs351855 (FGFR4)	Cyclophosphamide, fluorouracil, methotrexate	*A*/G	0.309	0.29	0.3029	1581	4.805E−01	6.06E−01
rs7136446 (IGF1)	tamoxifen	*T*/C	0.6372	0.61	NA	1589	5.265E−01	NA
rs1801133 (CLCN6,MTHFR)	Cyclophosphamide, fluorouracil	*A*/G	0.3674	0.35	0.345	1584	5.661E−01	7.67E−02
rs1045642 (ABCB1)	Idarubicin, taxanes, cyclophosphamide, fluorouracil, epirubicin, anthracyclines and related substances, doxorubicin, paclitaxel, cytarabine, tamoxifen	*G*/A	0.4894	0.47	0.4719	331	6.992E−01	6.17E−01
rs717620 (ABCC2)	Tamoxifen	*T*/C	0.2009	0.21	0.1993	331	7.801E−01	9.71E−01
rs3740065 (ABCC2)	Tamoxifen	*G*/A	0.1163	0.11	NA	331	8.022E−01	NA
rs1800566 (NQO1)	Fluorouracil, epirubicin doxorubicin, cyclophosphamide	*A*/G	0.2044	0.2	0.188	1590	8.642E−01	6.10E−02
rs4646 (CYP19A1)	Letrozole	*C*/A	0.7221	0.71	0.7481	331	8.697E−01	5.78E−01
rs9322336 (ESR1)	Exemestane	*T*/C	0.7825	0.77	NA	331	8.729E−01	NA
rs8060157 (ZNF423)	Tamoxifen, raloxifene	*G*/A	0.5548	0.56	NA	1258	9.202E−01	NA
rs1056892 (CBR3)	Doxorubicin	*A*/G	0.3566	0.36	0.3481	1590	9.352E−01	5.05E−01
rs10509681 (CYP2C8)	Paclitaxel	*C*/T	0.1086	0.11	0.1132	1589	9.719E−01	4.89E−01
rs20572 (CBR1, SETD4)	Doxorubicin, doxorubicinol	*T*/C	0.0997	0.1	0.1144	331	1.00	3.18E−01

Out of 41 variants selected for the analysis, thirteen of them (in italics) were found as significantly different in frequency in our sample as compared to the EUR cohort

*Snp id* variant name, *Gene* target gene, *Drugs* medication reported to be correlated with a variant/gene in PharmGkB database (https://www.pharmgkb.org/), *Alleles* variant alleles (tested allele in italics), *Af_FVG* allele frequency of the FVG population, *Af_1000G_EUR* allele frequency of 1000G European population, *Af_ExAC_NFE* allele frequency in ExAC Non Finnish European populations, *Samples* sample number, *p value vs EUR* p value from Fisher test versus EUR population from 1000G (total sample size 379), *p value vs ExAC* p-value from Fisher test versus ExAC populations (total sample size 33,368)

### Comparison between populations

All variants reported to be related to the efficacy or toxicity of breast cancer drugs were extracted from PharmGKB database (https://www.pharmgkb.org/ [14]), obtaining a set of 80 single nucleotide polymorphisms (SNPs), all located on autosomal chromosomes. The overlap between these data and our population’s genotypes resulted in two catalogs of 37 and 23 loci (respectively from joint dataset and HumanExome chip) for a unique list of 41 variants. Among these 41 variants we extracted frequency information in the general European cohort from 379 people of the 1000G Project data (EUR population) [15], 33,368 people (Non Finnish European individuals) from the ExAC database and [16] 1590 individuals from the FVG population using PLINK software [17].

For each variant, differences in allele frequencies between the three populations were tested by Fisher’s exact test. Due to the high number of people included in ExAC database compared to 1000G data, the estimation of frequencies is more precise thus leading to a higher significance p value. Because of the explorative nature of our analysis, no multiple testing corrections were performed, and a p value ≤0.05 was considered statistically significant.

### Aggregated analysis of risk variants

In order to estimate the overall genotype’s risk for therapeutical agents, six main classes of drugs were defined as follows: (1) fluoropyrimidines, (2) alkylating agents, (3) taxanes, (4) antracyclines/tumor antibiotics, (5) anti-estrogens, and (6) monoclonal antibodies. For each class of drugs, all the variants associated with breast cancer medications and having a different frequency between the FGV and the EUR/ExAC populations, were pooled and the proportion of the “at risk” genotypes in our population was defined.

## Results

A cohort of 1590 individuals from six different isolated villages located in FVG region was used as the base for the study (Fig. 1 and Additional file 1: Table S1). Out of 80 variants and 58 genes related to breast cancer medications according to the PharmGKB database, 41 variants in 32 genes were present in our genotyping platforms for the six isolated populations (Additional file 2: Table S2 and Fig. 2). The frequencies of 13 out of 41 variants (i.e., 32 %) were different between FGV and EUR/ExAC cohorts (Table 1). When available, level of evidence (LOE) of the variant-drug combination, scoring from 1 (annotation for which pharmacogenomics guidelines are implemented in clinical practice) to 4 (annotation based on a case report, non-significant study or in vitro, molecular or functional assay evidence only), is reported in Table 2. A more detailed description of these differences and the relative clinical impact is described in the following sections. We will first describe differences for those SNPs for which an association with activity or toxicity has been reported (LOE 1–4), see Table 2A. Then, we will discuss differences for the SNPs that were reported as “related” with breast cancer medications but for each the evidence supporting the association is lacking (LOE not reported; Table 2B). Finally, we will present the cumulative frequency of at risk genotype for the defined categories of drugs.

**Table 2:** Genotype’s counts for the 13 variants described as related to breast cancer medications in Italian isolated populations

Snp Id (gene)	Functional annotation	Level of evidence	Drug^a^	Minor	Major	Parameters	Risk allele	Genotype (A1A1-A1A2-A2A2)
A								
rs4244285 (*CYP2C19*)	exonic, synonymous	4	Doxorubicin, *cyclophosphamide*, tamoxifen	A	G	Efficacy	A (FVG freq <1000G/ExAC)	3AA-48AG-265GG
rs714368 (*SLC22A16* **)**	exonic, nonsynonymous	4	*Doxorubicin*, doxorubicinol, cyclophosphamide	C	T	Toxicity	T (FVG freq >1000G/ExAC)	118CC-618CT-836TT
rs4880 (*SOD2*)	exonic, nonsynonymous	2B/3*	*Cyclophosphamide/tamoxifen*	A	G	Efficacy	G (FVG freq >1000G/ExAC)	110GG-157GA-49AA
rs1801274 (*FCGR2A*)	exonic, nonsynonymous	2B	*Trastuzumab*, doxorubicin, paclitaxel, cyclophosphamide	G	A	Efficacy	G (FVG freq <1000G/ExAC)	289GG-741GA-542AA
rs1136201 (*ERBB2*)	exonic, nonsynonymous	3	*Trastuzumab*	G	A	Toxicity	G (FVG freq <1000G/ExAC)	49GG - 437GA - 1095AA
rs2297595 (*DPYD*)	exonic, nonsynonymous	2A	*Capecitabine, fluorouracil*	C	T	Toxicity	C (FVG freq <1000G/ExAC)	20CC-247CT-1314TT
rs3918290 (*DPYD*)	splicing, NA	1	*Capecitabine, fluorouracil*	T	C	Toxicity	T (FVG freq <1000G/ExAC)	0TT-2TC-1579CC
rs1048943 (*CYP1A1*)	exonic, nonsynonymous	3	Docetaxel, *capecitabine*	C	T	Efficacy	T (FVG freq >1000G/ExAC)	6CC-189CT-1386TT
rs776746^b^ (*CYP3A5*)	splicing, NA	3/Not reported	*Paclitaxel/tamoxifene*	T	C	Toxicity	T (FVG freq >1000G)	18TT-216TC-1337CC
B								
rs2369049 (*TCL1A*)	intergenic, NA	*Not reported*	*Exemestane, anastrozole*	G	A	Not defined	Not defined	18GG-315GA-1247AA
rs2072671 (*CDA*)	exonic, nonsynonymous	Not reported	Capecitabine	C	A	Not defined	Not defined	255CC-721CA-605AA
rs180 1159 (*DPYD*)	exonic, nonsynonymous	Not reported	Capecitabine	C	T	Not defined	Not defined	84CC-518CT-979TT
rs6214 (*IGF1*)	UTR3,NA	Not reported	Tamoxifen	T	C	Not defined	Not defined	198TT-707TC-666CC

A) variants that show significant difference in frequency between FVG sample set and EUR/ExAC populations in terms of level of association with drug response

B) variants that don’t show a level of evidence for drug response

As in most case the association has been evaluated in the context of polychemotherapeutic regimens, the most likely drug related to the target polymorphism is reported in italic

*Snp id* (*gene*) variant name, gene name, *Functional annotation* functional annotation for the variant, *Level of evidence* based on the amount of evidence reported at https://www.pharmgkb.org/that supports the association, *Drug* ^*a*^ medication reported to be correlated with a variant/gene after checking the literature thus filtering out the non-significative association reported in PharmGKB database, *Minor* minor allele, *Major* major allele, *Genotype* genotype’s count for FVG population

Most of the studies were conduced in the context of polychemitherapeutic regimens; in such studies the drugs most likely related to the targeted polymorphisms according to the available literature are highlighted in italics; *Parameters* reported by PharmaGKB database. *efficacy* poor outcome, *toxicity* significant side effects, *Risk allele* risk allele reported in literature.^b^ for variant rs776746 data for this variant was not available in the ExAC database at the time of the analysis, *Genotype* genotype’s counts

Level 1A: annotation for a variant-drug combination in a CPIC or medical society-endorsed PGx guideline, or implemented at a PGRN site or in another major health system; Level 1B: annotation for a variant-drug combination where the preponderance of evidence shows an association. The association must be replicated in more than one cohort with significant p-values, and preferably will have a strong effect size; Level 2A: annotation for a variant-drug combination that qualifies for level 2B where the variant is within a VIP (very important pharmacogene) as defined by PharmGKB. The variants in level 2A are in known pharmacogenes, so functional significance is more likely; Level 2B: annotation for a variant-drug combination with moderate evidence of an association. The association must be replicated but there may be some studies that do not show statistical significance, and/or the effect size may be small; Level 3: annotation for a variant-drug combination based on a single significant (not yet replicated) or annotation for a variant-drug combination evaluated in multiple studies but lacking clear evidence of an association; Level 4: annotation based on a case report, non-significant study or in vitro, molecular or functional assay evidence only

### Genetic variants associated to breast neoplasm medications

By using our analytical pipeline, we found nine variants associated with drug toxicity or efficacy having a different distribution between the FGV and the EUR/ExAC populations supported by a certain LOE (Table 2A). The level of association was reported as statistically significant in at least one study for seven of them (LOE 1–3) and borderline significant for one of them (rs4244285; LOE 4). In one case (rs714368), two studies reported opposite results (LOE 4), as described below (Table 2A). The type and the position of these variants are reported in Table 2A. Eight of them are missense variants, while two of them are annotated as splicing variants. For the remaining three one is a synonymous variant, one is located in UTR3 site and one is located in an intergenic region. A more detailed description of each variant including data on in silico prediction is reported in the Additional file 3: Table S3. Considering the variants that affect the splicing, one is reported to be a spice donor (rs3918290) and the other one (rs776746) as a splice acceptor located in the UTR5. In this light, a change in these sites could have functional consequences.

Doxorubicine and cyclophosphamide are the backbone of the chemotherapeutic regimens used for the treatment of breast cancer patients. Cyclophosphamide is a pro-drug that needs to be oxidized to exert its cytotoxic effect. This step is catalyzed by a number of cytochrome P450 enzymes, including *CYPC219* [18]. The cellular uptake of doxorubicine is mediated by *ABCB1* and *SLC22A16* cationic transporters [19].

As regards *CYP2C19*, the rs4244285 variant has been reported to be associated with differential response to cyclophosphamide–doxorubicin adjuvant regimen. The frequency of the A allele in the EUR/ExAC cohorts and in the mixed population is 15 % while in our cohort is 8 % (p = 1.1E−03) (Table 1). The GG genotype was the most frequent in FVG cohorts (with the highest number in Resia valley) (Table 2A). According to a single retrospective study [19], individuals bearing the AA genotype show a trend of an increased risk of poorer outcome if treated with cyclophosphamide–doxorubicin polychemotherapeutic regimen (LOE 4) although other studies are partially discordant [20].

As for *SLC22A16* gene, the rs714368 polymorphism, which is related to doxorubicin response, has a frequency of 35 % for the C allele in the Asian population; the frequency of this allele in the EUR/ExAC cohorts is 21–22 % while in our population is 27 % (p = 6.9E−03) (Table 1). A minor effect (p = 5.5E−2) of increased exposure to doxorubicin associated with rs714368 C rare allele homozygosity was observed in Lal et al. [21]. Conversely, a more recent study [19] has associated, a decreased incidence of dose delay with the carrying of the C allele, both at the homozygous and heterozygous status. Considering that the T allele is more frequent in FVG cohorts (Table 2A), there is a higher proportion of individuals which may experience higher toxicity for doxorubicin (LOE 4).

The rs4880 G allele (located within super superoxide dismutase 2-*SOD2*-gene) has been correlated with lower survival in breast cancer patients treated with chemotherapy (p = 1E−3). However, the effect was mostly restricted to those treated with cyclophosphamide-based adjuvant regimens (p value for cyclophosphamide–genotype interaction = 2.3E−2, LOE 2B), an association replicated in two independent cohorts (US and Norwegian patients) [22]. More recently, AA genotype has been associated with statistically significant better progression-free and overall survival in patients treated with adjuvant tamoxifen [23]. In the EUR/ExAC cohorts the frequency of the G allele is 46 and 51 % respectively while in FVG population is 54.5 % (p = 1.9E−02; 7.8E−02) suggesting a quite high percentage of at risk genotype in FVG region (Table 2A).

The introduction of the anti-HER2 monoclonal antibody (mAb) trastuzumab in clinical practice has revolutionized the treatment of HER-2 positive breast cancer patients. In fact, their mechanism of actions relies on their ability to inhibit the target surface molecules and to trigger antibody-dependent cellular cytotoxicity (ADCC), a process which involve fragment crystallizable (FC) receptors. Based on some retrospective studies, it could be speculated that polymorphisms of FC receptor resulting in differential FC affinity can reduce the activity of those mAb [24].

Regarding rs1801274 (*FCGR2A* gene), the frequency of G allele in our cohort is 42 % while in EUR/ExAC cohorts is 49–50 % (p = 1.5E−02; 9.2E−07) (Tables 1 and 2A). Exome chip data supports this result. The most frequent genotype in FVG cohorts is AG. In trastuzumab treated patients, this genotype, as compared to AA genotype, was significantly associated with decreased response and shorter progression-free survival (LOE 2B) [25, 26]. Nevertheless, these data should be considered with caution. In fact, the association between rs1801274 *FCGR2A* and response to trastuzumab in metastatic [25] or neoadjuvant [26] setting comes from the retrospective analyses of small patient cohorts. Conversely, Hurvitz et al. failed to reproduce these observations by evaluating more than one thousand patients enrolled onto an adjuvant randomized trial [24, 27].

Although trastuzumab is generally well tolerated, its use is often associated with a clinically relevant cardiotoxicity thus a particular caution in the qualification for treatment is necessary and the genotype information could improve the physician’s decision-making process. A recent investigation has shown that protein modification induced by rs1136201 *ERBB2* polymorphisms may render cardiomyocytes dependent upon *HER2* signaling and more sensitive to trastuzumab-mediated toxicity [28] (LOE 3). According to this study, patients with the AA genotype may have decreased risk of cardiotoxicity as compared to patients with the AG genotype following trastuzumab administration (p = 5.8E−3) [28]. The frequency of G allele for rs1136201 is 17 % in FVG cohorts and between 24 and 25 % in EUR/ExAC cohorts (p = 3.6E−5; 2.4E−13), while the frequency of low risk AA patients in our cohort is 69 % (Tables 1 and 2A).

Fluoropyrimidine (i.e., capecitabine, 5-fluoruracil) are another class of drugs widely used in breast cancer. Dihydropyrimidine dehydrogenase (DPD) eliminates more than 80 % of the administered drug and is the rate-limiting enzyme foruoropyrimidine catabolism. Cancer patients carrying mutations in the dihydropyrimidine dehydrogenase gene (*DPYD*) have a high risk to develop severe drug-adverse effects following fluoropyrimidine drugs administration. These side effects consist in myelosuppression, mucositis, neurotoxicity, hand–foot syndrome, and diarrhea, which can be life-threatening. Guidelines that recommend alternative drugs or different dose adjustment according to *DPYD* genotype have been developed [29, 30].

The *DPYD* rs2297595 C allele encodes for an inactive *DPYD* variant and has been strongly associated with fluoropyrimidine-related toxicity [10, 31, 32] (LOE 2A). The rs2297595 showed a frequency of C allele of 10–12 % in EUR/ExAC cohorts respectively while in our population is 7 % (p = 8.3E−3). Data on the extended sample size (1581 individuals) confirmed this result (allele C frequency = 9 %; p = 3.3E−02; 5.4E−02) (Tables 1 and 2A).

The rs3918290 is the most well studied *DPYD* variant and the T allele variant is associated with increased toxicity (LOE 1). This intronic polymorphism results in a splicing variant skipping an entire exon and a nonfunctional protein [33]). The frequency of T allele was extremely low in our population (0.063 % vs 0.6–1 %, FGV vs EUR/ExAC populations, respectively, Table 2A).

Homozygous TT genotypes (i.e., those with a severe outcome) are not present in our cohort, while there are two out of 1581 CT heterozygous individuals (five heterozygous cases out of 379 samples in EUR cohort). A low frequency of at risk *DPYD* rs2297595 C and rs3918290 T genotypes might be a good indicator for physicians (see Table 2A).

We also noticed a different distribution of rs1048943 (*CYP1A1* gene) in our vs EUR/ExAC cohort. *CYP1A1* is likely involved in the metabolism of fluoropyrimidine [34]. Metastatic breast cancer patients carrying TT genotype may suffer a decreased progression-free survival when treated with capecitabine plus docetaxel [35] (LOE 3). The frequency of C allele in our cohort was 6.8 % (6.3 % in Exome chip data) and 3–4 % in the EUR/ExAC cohort (p = 2.4E−2, 5.7E−18). Although in the PharmGKB website is reported an association between this variant and taxane, Vaclavikova et al. indicates that *CYP1A1* does not metabolize taxanes [36], implying that the association noticed is driven by the effect on this polymorphism on capecitabine.

Finally, we found a different distribution of rs776746 (*CYP3A5*) T allele between EUR (8 %) and our population (5 %). In this regard, a recent study reported this variant as significantly associated with severe neutropenia in breast cancer patients treated with paclitaxel (LOE 3), another critical drug widely used in both metastatic and adjuvant setting [37] (Table 2A). This polymorphism has also been studied in the setting of adjuvant tamoxifen, but no correlation with recurrence risk of disease has been detected [38].

### Genetic variants with doubtful role related with breast cancer medications

In addition to the above mentioned associations, we found 4 variants (rs2369049 close to *TCL1A* gene, rs2072671 within *CDA* gene; rs1801159 within *DPYD* gene, rs6214 within *IGF1* gene) of genes listed as “related” to breast cancer medications by PharmGKB having a different distribution between the FGV and EUR/ExAC cohorts but for which the LOE of drug-variant association was not reported. By reviewing the pertinent literature we found that all but one of these polymorphisms (rs2369049-*TCL1A*), the underlying studies were largely negative [39, 40]. As for rs2369049, however, two studies detected an association with exemestane toxicity but in opposite direction [39, 41].

### Aggregated analysis of variants at risk

To further increase our knowledge on combined at risk genotypes (i.e., pooling together the genetic data available regarding) related to each treatment, we divided therapeutic agents in six main classes (see “Methods” section). The combined presence of the described risk alleles (Table 2A) was checked within each class.

For three of those classes, the members (i.e., the breast cancer medications) did not share any of the reported at risk genotype. We then defined, for the three remaining classes, the proportion of at risk subjects within each class by calculating the overall cumulative frequency of at risk genotypes (Table 3). The results are described as follows.

**Table 3:** At risk genotype’s counts by drug/therapy class

Class number	Drug class	At risk genotype	At risk genotype count	Samples	Genotype frequency
1	Fluoropyrimidines				
	rs2297595 + rs1048943 + rs3918290	CC + CT & TT & TT + CT	0	1581	0
	rs2297595 + rs1048943	CC + CT & TT	20	1581	1.26 %
	rs2297595 + rs3918290	CC + CT & TT + CT	1	1581	0.06 %
	rs1048943 + rs3918290	TT & TT + CT	1	1581	0.06 %
2	Alkylating agents				
	rs4880 + rs4244285	GA + GG & AA	4	331	1.20 %
3	Monoclonal antibodies				
	rs1136201 + rs1801274	GA & GG + GA	291	1581	18.40 %

Out of six classes, members of three of them (i.e., the breast cancer medications) did not share any of the reported at risk genotype. For class 1, three SNPs related with Fluoropyrimidines were found and different combinations are reported. *Drug class* class of drugs or treatment, *At risk genotype* risk genotype for the class, *At risk genotype count* count of joint risk genotypes for the class, Samples total number of analyzed samples, *Genotype frequency* at risk genotype frequency

Fluoropyrimidines (capecitabine and 5-fluorouracil). No subjects carried all the three at risk genotypes [e.g., rs2297595 (*DPYD* gene), rs1048943 (*CYP1A1* gene), and rs3918290 (*DPYD* gene)]. We observed that only 1.26 % of the analyzed samples carried both at risk genotypes for rs2297595 and rs1048943 polymorphisms, while the 0.06 % of subjects carried the rs3918290 and rs1048943 at risk alleles or the rs3918290 and rs2297595 risk alleles.

Alkylating agents (cyclophosphamide). Approximately 1 % (1.20 %) of our sample could be at risk of a poor outcome being a carrier of both at risk genotypes for rs4880 and rs4244285 variants.

Monoclonal antibodies (i.e., trastuzumab). Approximately 18 % (18.40 %) of our cohort carried the at risk genotypes for both rs1136201 and rs1801274.

## Discussion

In this study, by taking advantage of the participation to a Pilot National Project on a specific set of isolated communities of North-Eastern Italy (i.e., FVG population) and compared to those from EUR population and ExAC database, we described the distribution of polymorphisms related to breast cancer medications.

Despite the frequencies of most polymorphisms were similar between the EUR, ExAC and the FVG cohort, more than 30 % of variants analyzed significantly differed among these cohorts. A certain LOE was reported for 9 out of those 13 variants, while for four of them the related literature fails to detect any kind of association (LOE not reported).

Variants for which a LOE was available include polymorphisms linked to cyclophosphamide, tamoxifen, doxorubicin, fluorpyrimidine and paclitaxel. As for cyclophosphamide, one polymorphism (i.e., rs4880-G) within the *SOD2* gene was associated with poor outcome (LOE 2B) and showed a higher frequency in the FVG as compared to EUR/ExAC cohort. Another variant, located in the *CYP2C19* gene (i.e., rs4244285-A), had a lower frequency in our population. Importantly, the rs4880-G has also been associated with poor response to tamoxifen (LOE 3). Notably, only a small proportion of FVG population (i.e., 1.2 %) carried both at risk polymorphisms.

Regarding doxorubicine and paclitaxel, we observed that the frequency of two alleles such as rs714368-T (*SLC22A16*) and rs776746-C (*CYP3A5*), associated respectively with toxicity to doxorubicine (LOE 4) and paclitaxel (LOE 3), displayed a higher frequency in the FVG cohort. These findings imply that that those two drugs could be less tolerated by FVG patients.

As for trastuzumab, one variant within the *FCGR2A* gene (rs1801274-G), which is associated with decreased efficacy (LOE 2B), and one variant within the *ERBB2* gene, which is implicated in cardiomyopathy development (LOE 3), were underrepresented in FVG population suggesting an higher therapeutic index of this drug in our population as compared to that expected in the overall European population. Nevertheless, 18.4 % of subjects in our cohort carried the at risk genotypes for both rs1136201 and rs1801274, thus highlighting that a considerable proportion of patients in FVG displays a particularly unfavorable genotype despite the frequency of such polymorphisms is lower as compared to the one observed in the EUR cohort.

As far as fluorpyrimidines is concerned, the frequency of two polymorphisms within the *DPYD* gene associated with drug toxicity (e.g., rs2297595 C allele and rs3918290 T allele, LOE 2A and 1, respectively) was extremely low in FVG population. A number of studies have conclusively demonstrated that patients with functional alteration of *DPYD* gene can experience life threatening adverse events following fluoropyrimidine administration. The rs3918290 is the most well characterized *DPYD* variant and dosing guidelines have been developed. The clinical pharmacogenetics implementation consortium (CPIC) for dihydropyrimidine dehydrogenase genotype and fluoropyrimidine dosing recommends to select alternative drugs in case of homozygosity for rs3918290 T allele and to reduce dose by 50 % or select alternative drug in case of heterozygosity [29]. T allele was extremely rare in FVG population and homozygous TT genotypes (i.e., those ones with a severe outcome) were not present in our cohort, while there were only 2 out of 1581 CT heterozygous individuals. The frequency of the rs1048943 T allele (*CYP1A1* gene), which has been associated with toxicity of capecitabine-containg regimens, was higher in FVG population. However, the clinical relevance of this association is not clearly defined (LOE 3). Importantly, only 0.06 % of the FVG patients carried both at risk genotypes for rs3918290 and rs1048943 polymorphisms and only 1.2 % for rs2297595 and rs1048943 variants. Overall, considering the clinical relevance of the *DPYD* data, these results suggest that a larger number of FVG patients could benefit from full dosage of fluoropyrimidine therapy.

As for tamoxifene, several studies have assessed the impact of cytochrome *CYP2D6* genotype on treatment responsiveness but results are clashing. Although *CYP2D6* data were not available in our cohort, a recent meta-analysis on 25 studies enrolling more than 13 thousand individuals concluded that there is no sufficient evidence to support *CYP2D6* genotyping in patients treated with tamoxifen [40].

The information derived from our study will be transferred to the Regional Health Care Network in order to prepare specific leaflets to accurately inform local hospitals and physicians allowing the implementation of genomic medicine. While we found that one-third of the analyzed variants had a different frequency in our vs the EUR/ExAC cohort, we also noticed that for most of the assessed targets the respective LOE was weak (LOE 3–4) and further investigations are needed to confirm the reported associations. Implementation of such approach in breast-cancer clinical setting could fill this knowledge gap, which is a necessary step to prospectively refine the impact of a patient-based personalized treatment.

## Conclusions

In conclusion, our explorative study highlights the importance of assessing gene polymorphisms related with cancer medications in isolated populations. In particular, the finding that specific functional variants, strongly associated with toxicity or lack of efficacy, are more prevalent in a specific community could lead to the development of regional targeted interventions aimed at a direct screening of such risk genes/variants for example by using targeted re-sequencing approach. This in turn could facilitate a more effective and rationale usage of the healthcare economic resources thus paving the way for a personalized medicine [42].

## Additional files

10.1186/s12967-016-0778-z Demographic information about FVG population. Village: village to which the samples belong; Participants: number of samples; Males: males number; Females (%): females number (percentage); Mean Age (SD): mean age in each village and its standard deviation.
10.1186/s12967-016-0778-z. List of variants and genes reported as associated with breast cancer medications by the PharmGKB website (https://www.pharmgkb.org/). In bold are reported the variants analysed in FVG population being available in our genotyping platforms. Drugs: breast cancer medication reported with a variant/gene in PharmGkB database (http://www.pharmgkb.org/); Snp Id: Single nucleotide polimorphism variant name; Gene: target gene; Alleles: alleles of target variant; Chip: genotyping platform available.
10.1186/s12967-016-0778-z Consequence annotation for analyzed variants from ANNOVAR.

## Abbreviations

FVG: Friuli Venezia Giulia
EUR: European population
ExAC: ExAC database

## References

[1] Martincorena I, Campbell PJ (2015). Somatic mutation in cancer and normal cells. Science.

[2] Ma Q, Lu AYH (2011). Pharmacogenetics, pharmacogenomics, and individualized medicine. Pharmacol Rev.

[3] Berry D (2005). Breast cancer heterogeneity may explain peaks in recurrence. Int J Surg Lond Engl.

[4] Peto R, Davies C, Godwin J, Gray R, Pan HC, Clarke M, Cutter D, Darby S, McGale P, Taylor C, Wang YC, Bergh J, Di Leo A, Albain K, Swain S, Piccart M, Pritchard K, Early Breast Cancer Trialists’ Collaborative Group(EBCTCG) (2012). Comparisons between different polychemotherapy regimens for early breast cancer: meta-analyses of long-term outcome among 100,000 women in 123 randomised trials. Lancet.

[5] Bedognetti D, Sertoli MR, Pronzato P, Del Mastro L, Venturini M, Taveggia P, Zanardi E, Siffredi G, Pastorino S, Queirolo P, Gardin G, Wang E, Monzeglio C, Boccardo F, Bruzzi P (2011). Concurrent vs sequential adjuvant chemotherapy and hormone therapy in breast cancer: a multicenter randomized phase III trial. J Natl Cancer Inst.

[6] Buyse M, Michiels S (2013). Omics-based clinical trial designs. Curr Opin Oncol.

[7] Ascierto ML, Idowu MO, Zhao Y, Khalak H, Payne KK, Wang X-Y, Dumur CI, Bedognetti D, Tomei S, Ascierto PA, Shanker A, Bear HD, Wang E, Marincola FM, De Maria A, Manjili MH (2013). Molecular signatures mostly associated with NK cells are predictive of relapse free survival in breast cancer patients. J Transl Med.

[8] Bedognetti D, Hendrickx W, Marincola FM, Miller LD (2015). Prognostic and predictive immune gene signatures in breast cancer. Curr Opin Oncol.

[9] Han X, Diao L, Xu Y, Xue W, Ouyang T, Li J, Wang T, Fan Z, Fan T, Lin B, Xie Y (2014). Association between the HER2 Ile655Val polymorphism and response to trastuzumab in women with operable primary breast cancer. Ann Oncol Off J Eur Soc Med Oncol ESMO.

[10] Deenen MJ, Tol J, Burylo AM, Doodeman VD, de Boer A, Vincent A, Guchelaar H-J, Smits PHM, Beijnen JH, Punt CJA, Schellens JHM, Cats A (2011). Relationship between single nucleotide polymorphisms and haplotypes in DPYD and toxicity and efficacy of capecitabine in advanced colorectal cancer. Clin Cancer Res Off J Am Assoc Cancer Res.

[11] Westbrook K, Stearns V (2013). Pharmacogenomics of breast cancer therapy: an update. Pharmacol Ther.

[12] Tabor HK, Auer PL, Jamal SM, Chong JX, Yu J-H, Gordon AS, Graubert TA, O’Donnell CJ, Rich SS, Nickerson DA, Bamshad MJ, NHLBI Exome Sequencing Project (2014). Pathogenic variants for Mendelian and complex traits in exomes of 6,517 European and African Americans: implications for the return of incidental results. Am J Hum Genet.

[13] Esko T, Mezzavilla M, Nelis M, Borel C, Debniak T, Jakkula E, Julia A, Karachanak S, Khrunin A, Kisfali P, Krulisova V, Aušrelé Kučinskiené Z, Rehnström K, Traglia M, Nikitina-Zake L, Zimprich F, Antonarakis SE, Estivill X, Glavač D, Gut I, Klovins J, Krawczak M, Kučinskas V, Lathrop M, Macek M, Marsal S, Meitinger T, Melegh B, Limborska S, Lubinski J (2013). Genetic characterization of northeastern Italian population isolates in the context of broader European genetic diversity. Eur J Hum Genet.

[14] The Pharmacogenomics Knowledge Base. [https://www.pharmgkb.org]. Accessed 18 Oct 2015.

[15] Consortium T 1000 GP (2010). A map of human genome variation from population-scale sequencing. Nature.

[16] Exome Aggregation Consortium (ExAC),Cambridge, MA. [http://exac.broadinstitute.org] Accessed 7 Dec 2015.

[17] Purcell S, Neale B, Todd-Brown K, Thomas L, Ferreira MAR, Bender D, Maller J, Sklar P, de Bakker PIW, Daly MJ, Sham PC (2007). PLINK: a tool set for whole-genome association and population-based linkage analyses. Am J Hum Genet.

[18] Jamieson D, Lee J, Cresti N, Jackson R, Griffin M, Sludden J, Verrill M, Boddy AV (2014). Pharmacogenetics of adjuvant breast cancer treatment with cyclophosphamide, epirubicin and 5-fluorouracil. Cancer Chemother Pharmacol.

[19] Bray J, Sludden J, Griffin MJ, Cole M, Verrill M, Jamieson D, Boddy AV (2010). Influence of pharmacogenetics on response and toxicity in breast cancer patients treated with doxorubicin and cyclophosphamide. Br J Cancer.

[20] Tulsyan S, Agarwal G, Lal P, Mittal B (2014). Significant role of CYP450 genetic variants in cyclophosphamide based breast cancer treatment outcomes: a multi-analytical strategy. Clin Chim Acta.

[21] Lal S, Wong ZW, Jada SR, Xiang X, Chen Shu X, Ang PCS, Figg WD, Lee EJ, Chowbay B (2007). Novel SLC22A16 polymorphisms and influence on doxorubicin pharmacokinetics in Asian breast cancer patients. Pharmacogenomics.

[22] Glynn SA, Boersma BJ, Howe TM, Edvardsen H, Geisler SB, Goodman JE, Ridnour LA, Lønning PE, Børresen-Dale A-L, Naume B, Kristensen VN, Chanock SJ, Wink DA, Ambs S (2009). A mitochondrial target sequence polymorphism in manganese superoxide dismutase predicts inferior survival in breast cancer patients treated with cyclophosphamide. Clin Cancer Res Off J Am Assoc Cancer Res.

[23] Tengström M, Mannermaa A, Kosma V-M, Soini Y, Hirvonen A, Kataja V (2014). MnSOD rs4880 and XPD rs13181 polymorphisms predict the survival of breast cancer patients treated with adjuvant tamoxifen. Acta Oncol.

[24] Mellor JD, Brown MP, Irving HR, Zalcberg JR, Dobrovic A (2013). A critical review of the role of Fc gamma receptor polymorphisms in the response to monoclonal antibodies in cancer. J Hematol OncolJ Hematol Oncol.

[25] Musolino A, Naldi N, Bortesi B, Pezzuolo D, Capelletti M, Missale G, Laccabue D, Zerbini A, Camisa R, Bisagni G, Neri TM, Ardizzoni A (2008). Immunoglobulin G fragment C receptor polymorphisms and clinical efficacy of trastuzumab-based therapy in patients with HER-2/neu-positive metastatic breast cancer. J Clin Oncol Off J Am Soc Clin Oncol.

[26] Tamura K, Shimizu C, Hojo T, Akashi-Tanaka S, Kinoshita T, Yonemori K, Kouno T, Katsumata N, Ando M, Aogi K, Koizumi F, Nishio K, Fujiwara Y (2011). FcγR2A and 3A polymorphisms predict clinical outcome of trastuzumab in both neoadjuvant and metastatic settings in patients with HER2-positive breast cancer. Ann Oncol Off J Eur Soc Med Oncol ESMO.

[27] Hurvitz SA, Betting DJ, Stern HM, Quinaux E, Stinson J, Seshagiri S, Zhao Y, Buyse M, Mackey J, Driga A, Damaraju S, Sliwkowski MX, Robert NJ, Valero V, Crown J, Falkson C, Brufsky A, Pienkowski T, Eiermann W, Martin M, Bee V, Marathe O, Slamon DJ, Timmerman JM (2012). Analysis of Fcγ receptor IIIa and IIa polymorphisms: lack of correlation with outcome in trastuzumab-treated breast cancer patients. Clin Cancer Res Off J Am Assoc Cancer Res.

[28] Beauclair S, Formento P, Fischel JL, Lescaut W, Largillier R, Chamorey E, Hofman P, Ferrero JM, Pagès G, Milano G (2007). Role of the HER2 [Ile655Val] genetic polymorphism in tumorogenesis and in the risk of trastuzumab-related cardiotoxicity. Ann Oncol Off J Eur Soc Med Oncol ESMO.

[29] Caudle KE, Thorn CF, Klein TE, Swen JJ, McLeod HL, Diasio RB, Schwab M (2013). Clinical pharmacogenetics implementation consortium guidelines for dihydropyrimidine dehydrogenase genotype and fluoropyrimidine dosing. Clin Pharmacol Ther.

[30] Swen JJ, Wilting I, de Goede AL, Grandia L, Mulder H, Touw DJ, de Boer A, Conemans JMH, Egberts TCG, Klungel OH, Koopmans R, van der Weide J, Wilffert B, Guchelaar H-J, Deneer VHM (2008). Pharmacogenetics: from bench to byte. Clin Pharmacol Ther.

[31] Gross E, Busse B, Riemenschneider M, Neubauer S, Seck K, Klein H-G, Kiechle M, Lordick F, Meindl A (2008). Strong association of a common dihydropyrimidine dehydrogenase gene polymorphism with fluoropyrimidine-related toxicity in cancer patients. PLoS One.

[32] Gross E, Ullrich T, Seck K, Mueller V, de Wit M, von Schilling C, Meindl A, Schmitt M, Kiechle M (2003). Detailed analysis of five mutations in dihydropyrimidine dehydrogenase detected in cancer patients with 5-fluorouracil-related side effects. Hum Mutat.

[33] Van Kuilenburg AB, Vreken P, Beex LV, Meinsma R, Van Lenthe H, De Abreu RA, van Gennip AH (1990). Heterozygosity for a point mutation in an invariant splice donor site of dihydropyrimidine dehydrogenase and severe 5-fluorouracil related toxicity. Eur J Cancer Oxf Engl.

[34] Choi YH, Bae SK, Kim SO, Lee MG (2007). Pharmacokinetics of 5-fluorouracil in mutant Nagase analbuminemic rats: faster metabolism of 5-fluorouracil via CYP1A. Biopharm Drug Dispos.

[35] Dong N, Yu J, Wang C, Zheng X, Wang Z, Di L, Song G, Zhu B, Che L, Jia J, Jiang H, Zhou X, Wang X, Ren J (2012). Pharmacogenetic assessment of clinical outcome in patients with metastatic breast cancer treated with docetaxel plus capecitabine. J Cancer Res Clin Oncol.

[36] Vaclavikova R, Soucek P, Svobodova L, Anzenbacher P, Simek P, Guengerich FP, Gut I (2004). Different in vitro metabolism of paclitaxel and docetaxel in humans, rats, pigs, and minipigs. Drug Metab Dispos Biol Fate Chem.

[37] Tang NLS, Liao CD, Wang X, Mo FKF, Chan VTC, Ng R, Pang E, Suen JJS, Woo J, Yeo W (2012). Role of pharmacogenetics on adjuvant chemotherapy-induced neutropenia in Chinese breast cancer patients. J Cancer Res Clin Oncol.

[38] Sensorn I, Sirachainan E, Chamnanphon M, Pasomsub E, Trachu N, Supavilai P, Sukasem C, Pinthong D (2013). Association of CYP3A4/5, ABCB1 and ABCC2 polymorphisms and clinical outcomes of Thai breast cancer patients treated with tamoxifen. Pharmacogenomics Pers Med.

[39] Ingle JN, Schaid DJ, Goss PE, Liu M, Mushiroda T, Chapman J-AW, Kubo M, Jenkins GD, Batzler A, Shepherd L, Pater J, Wang L, Ellis MJ, Stearns V, Rohrer DC, Goetz MP, Pritchard KI, Flockhart DA, Nakamura Y, Weinshilboum RM (2010). Genome-Wide Associations and Functional Genomic Studies of Musculoskeletal Adverse Events in Women Receiving Aromatase Inhibitors. J Clin Oncol..

[40] Lum DWK, Perel P, Hingorani AD, Holmes MV (2013). CYP2D6 genotype and tamoxifen response for breast cancer: a systematic review and meta-analysis. PLoS One.

[41] Henry NL, Skaar TC, Dantzer J, Li L, Kidwell K, Gersch C, Nguyen AT, Rae JM, Desta Z, Oesterreich S, Philips S, Carpenter JS, Storniolo AM, Stearns V, Hayes DF, Flockhart DA (2013). Genetic associations with toxicity-related discontinuation of aromatase inhibitor therapy for breast cancer. Breast Cancer Res Treat.

[42] Huttin CC, Liebman MN (2013). The economics of biobanking and pharmacogenetics databasing: the case of an adaptive platform on breast cancer. Technol Health Care Off J Eur Soc Eng Med.

