# Supplementary material for: Pharmacogenetics driving personalized medicine: analysis of genetic polymorphisms related to breast cancer medications in Italian isolated populations
Source: J Transl Med. 2016 Jan 22;14:22. doi: 10.1186/s12967-016-0778-z (PMC4722680; doi:10.1186/s12967-016-0778-z)
Supplement: Supplementary file 1 — 10.1186/s12967-016-0778-z Demographic information about FVG population. Village: village to which the samples belong; Participants: number of samples; Males: males number; Females (%): females number (percentage); Mean Age (SD): mean age in each village and its standard deviation. [file 12967_2016_778_MOESM1_ESM.docx]

| **Village** | **Participants** | **Males** | **Females (%)** | **Mean age (sd)** |
| --- | --- | --- | --- | --- |
| Clauzetto | 188 | 73 | 115 (61.17) | 47.62 (26.84) |
| Erto-Casso | 235 | 97 | 138 (58.72) | 46.09 (22.65) |
| Illegio | 218 | 89 | 129 (59.17) | 44.56 (22.29) |
| Resia | 551 | 230 | 321 (58.25) | 50.77 (18.12) |
| San Martino del Carso | 207 | 106 | 101 (48.79) | 49.01 (19.17) |
| Sauris | 191 | 84 | 107 (56.02) | 49.86 (24.28) |

**Tab.S1: Demographic information about FVG population**

**Village**: village to which the samples belong; **Participants**: number of samples; **Males**: males number; **Females (%)**: females number (percentage); **Mean Age (SD)**: mean age in each village and its standard deviation
