# Supplementary material for: Pharmacogenetics driving personalized medicine: analysis of genetic polymorphisms related to breast cancer medications in Italian isolated populations
Source: J Transl Med. 2016 Jan 22;14:22. doi: 10.1186/s12967-016-0778-z (PMC4722680; doi:10.1186/s12967-016-0778-z)
Supplement: Supplementary file 2 — 10.1186/s12967-016-0778-z. List of variants and genes reported as associated with breast cancer medications by the PharmGKB website (https://www.pharmgkb.org/). In bold are reported the variants analysed in FVG population being available in our genotyping platforms. Drugs: breast cancer medication reported with a variant/gene in PharmGkB database (http://www.pharmgkb.org/); Snp Id: Single nucleotide polimorphism variant name; Gene: target gene; Alleles: alleles of target variant; Chip: genotyping platform available. [file 12967_2016_778_MOESM2_ESM.docx]

| **Drugs (reported as related by PharmGKB database)** | **Snp Id** | **Gene** | **Alleles** | **Chip** |
| --- | --- | --- | --- | --- |
| anthracyclines and related substances,paclitaxel,  doxorubicin,  cyclophosphamide,taxanes | **rs2032582** | ***ABCB1*** | **A>C,A>T** | **700K,ExomeChip** |
| caffeine | rs762551 | *CYP1A2* | C>A | #N/A |
| capecitabine | rs11075646 | *CES2* | C>G | #N/A |
|  | **rs1801159** | ***DPYD*** | **T>C** | **700K,ExomeChip** |
|  | **rs2072671** | ***CDA*** | **A>C,A>T,A>G** | **300K,700K,**  **ExomeChip** |
|  | **rs2290272** | ***SLC28A1*** | **G>A** | **300K,700K,**  **ExomeChip** |
|  | **rs3918290** | ***DPYD*** | **C>T** | **ExomeChip** |
| carboplatin,gemcitabine | rs34489327 | *ENOSF1,TYMS* | T>TTAAAG,T>- | #N/A |
| cyclophosphamide | **rs4880** | ***SOD2*** | **A>G** | **700K,ExomeChip** |
| cyclophosphamide,  fluorouracil | **rs1801133** | ***CLCN6,MTHFR*** | **G>A** | **300K,700K,**  **ExomeChip** |
|  | rs34743033 | *TYMS* | CCGCGCCACTTGGCCTG>4,  CCGCGCCACTTGGCCTG>3,  CCGCGCCACTTGGCCTG>? | #N/A |
| cyclophosphamide,  fluorouracil,methotrexate | **rs351855** | ***FGFR4*** | **G>A** | **700K,ExomeChip** |
|  | **rs45589337** | ***DPYD*** | **T>C** | **ExomeChip** |
| docetaxel,  capecitabine | **rs1048943** | ***CYP1A1*** | **A>C,A>T,A>G** | **700K,ExomeChip** |
| docetaxel,epirubicin,  fluorouracil,doxorubicin,  cyclophosphamide | **rs1695** | ***GSTP1*** | **A>G** | **300K,700K,**  **ExomeChip** |
| docetaxel,paclitaxel,taxanes | **rs1056836** | ***CYP1B1*** | **C>G** | **700K,ExomeChip** |
| doxorubicin | **rs1056892** | ***CBR3*** | **G>A** | **300K,700K,**  **ExomeChip** |
|  | **rs1128503** | ***ABCB1*** | **A>G** | **700K** |
|  | **rs8133052** | ***CBR3*** | **G>A** | **300K,700K** |
| doxorubicin,  cyclophosphamide | **rs1143684** | ***NQO2*** | **C>T** | **300K,700K,**  **ExomeChip** |
|  | **rs12210538** | ***SLC22A16*** | **A>G** | **700K,ExomeChip** |
|  | rs12721655 | *CYP2B6* | A>G | #N/A |
|  | rs3211371 | *CYP2B6* | C>T | #N/A |
|  | rs3745274 | *CYP2B6* | G>T | #N/A |
|  | **rs4244285** | ***CYP2C19*** | **G>C,G>A** | **700K** |
|  | rs6907567 | *SLC22A16* | A>G | #N/A |
|  | **rs723685** | ***SLC22A16*** | **A>G** | **300K,700K,**  **ExomeChip** |
|  | rs8292709 | *CYP2B6* | C>T | #N/A |
| doxorubicin,  cyclophosphamide,  fluorouracil | **rs9561778** | ***ABCC4*** | **G>T,G>A** | **700K** |
| doxorubicin,  cyclophosphamide,  fluorouracil,methotrexate | rs1799983 | *NOS3* | T>G | #N/A |
|  | rs2070744 | *NOS3* | C>T | #N/A |
| doxorubicin,docetaxel | rs1937840 | *AKR1C3* | C>G | #N/A |
| doxorubicin,doxorubicinol | **rs20572** | ***CBR1,SETD4*** | **C>T** | **700K** |
|  | **rs714368** | ***SLC22A16*** | **T>C** | **300K,700K** |
|  | **rs9024** | ***CBR1,SETD4*** | **G>A** | **300K,700K** |
| estradiol | rs1864729 | *TSPYL5* | G>A | #N/A |
| exemestane | rs11849538 | *TCL1A* | C>G | #N/A |
|  | **rs2369049** | ***TCL1A*** | **A>G** | **ExomeChip** |
|  | **rs9322336** | ***ESR1*** | **C>T** | **700K** |
| exemestane,  anastrozole,letrozole | rs6493497 | *CYP19A1*  *,GLDN* | G>A | #N/A |
|  | rs7176005 | *CYP19A1,*  *GLDN* | C>T | #N/A |
| fluorouracil,capecitabine | **rs2297595** | ***DPYD*** | **T>C** | **700K,ExomeChip** |
| fluorouracil,  epirubicin,doxorubicin,  cyclophosphamide | **rs1800566** | ***NQO1*** | **C>G,C>A** | **300K,700K,**  **ExomeChip** |
| fluorouracil,paclitaxel,  antineoplastic agents,  cyclophosphamide,cisplatin | **rs1042522** | ***TP53,WRAP53*** | **C>G** | **700K** |
| fluorouracil,paclitaxel,  doxorubicin,  cyclophosphamide,tamoxifen | rs2740574 | *CYP3A4* | C>T | #N/A |
| gemcitabine | rs1042658 | *RRM1* | G>A | #N/A |
|  | rs9937 | *RRM1* | A>G | #N/A |
| idarubicin,taxanes,  cyclophosphamide,  fluorouracil,epirubicin,  anthracyclines and related substances,doxorubicin,  paclitaxel,cytarabine,  tamoxifen | **rs1045642** | ***ABCB1*** | **A>T,A>G** | **700K** |
| lapatinib | rs17852153 | *CCND1* | NA | #N/A |
| letrozole | **rs4646** | ***CYP19A1*** | **A>C** | **700K** |
| Multivitamins,plain | rs70991108 | *DHFR* | ->TGGCGCGTCCCG | #N/A |
| paclitaxel | **rs10509681** | ***CYP2C8*** | **T>C** | **300K,700K,**  **ExomeChip** |
|  | rs10771973 | *FGD4* | G>A | #N/A |
|  | rs7001034 | *FZD3* | A>G | #N/A |
|  | **rs7349683** | ***EPHA5*** | **C>T** | **300K,700K** |
| procarbazine,dacarbazine | rs1800734 | *EPM2AIP1* | G>A | #N/A |
| tamoxifen | **rs10509373** | ***C10orf11*** | **T>C** | **300K,700K** |
|  | rs1065852 | *CYP2D6* | C>G,C>A | #N/A |
|  | rs11023197 | *RRAS2* | G>A | #N/A |
|  | rs1135840 | *CYP2D6* | G>C | #N/A |
|  | rs12248560 | *CYP2C19* | C>T,C>A | #N/A |
|  | rs16947 | *CYP2D6* | A>G | #N/A |
|  | rs1801123 | *IRS1* | T>C | #N/A |
|  | rs2016347 | *IGF1R* | G>T | #N/A |
|  | rs28371725 | *CYP2D6* | C>T | #N/A |
|  | rs2854744 | *IGFBP3* | G>T | #N/A |
|  | rs2946834 | *IGF1* | A>G | #N/A |
|  | rs3740065 | *ABCC2* | A>G | 700K |
|  | rs3892097 | *CYP2D6* | C>T | #N/A |
|  | **rs6214** | ***IGF1*** | **C>T** | **300K,700K** |
|  | **rs7136446** | ***IGF1*** | **C>T** | **300K,700K** |
|  | **rs717620** | ***ABCC2*** | **C>T** | **700K** |
|  | **rs776746** | ***CYP3A,CYP3A5*** | **C>T** | **300K,700K** |
|  | rs9282861 | *SULT1A1,*  *SULT1A2* | G>C,G>T | #N/A |
|  | rs9340799 | *ESR1* | A>G | #N/A |
| tamoxifen,raloxifene | rs10030044 |  | G>T | #N/A |
|  | **rs8060157** | ***ZNF423*** | **A>G** | **300K,700K** |
| trastuzumab | **rs1136201** | ***ERBB2*** | **A>T,A>G** | **ExomeChip** |
|  | **rs396991** | ***FCGR3A*** | **A>C** | **#N/A** |
| trastuzumab,doxorubicin,  paclitaxel,cyclophosphamide | **rs1801274** | ***FCGR2A*** | **A>G** | **300K,700K,**  **ExomeChip** |

**Table s1**: **List of variants and genes reported as associated with breast cancer medications by the PharmGKB website (**[**https://www.pharmgkb.org/**](https://www.pharmgkb.org/)**).**

**In bold are reported the variants analysed in FVG population** being available in our genotyping platforms.

**Drugs**: breast cancer medication reported with a variant/gene in PharmGkB database (http://www.pharmgkb.org/); **Snp Id**: Single Nucleotide Polimorphism variant name; **Gene**: target gene; **Alleles**: alleles of target variant; **Chip**: genotyping platform available
